# Cross-Cultural Measurement Invariance of a Measure of Disability for White, Black, Hispanic and Asian Older Adults

**DOI:** 10.3390/ijerph18041401

**Published:** 2021-02-03

**Authors:** Keith T. Chan, Carl Algood, Andreana Prifti, Tarek Zidan

**Affiliations:** 1 Silberman School of Social Work at Hunter College, The City University of New York, 2180 3rd Ave, New York, NY 10035, USA; 2 School of Social Work, University of Maryland Baltimore, Baltimore, MD 21201, USA; algoodcl@gmail.com; 3 College of Arts & Sciences, University at Albany, State University of New York, Albany, NY 12222, USA; andrianaprifti@gmail.com; 4 School of Social Work, Indiana University in South Bend, South Bend, IN 46634, USA; tzidan@iu.edu

**Keywords:** disability, race, ethnicity, measurement, older adults

## Abstract

Introduction: This study aims to determine the cross-cultural measurement equivalence of the Washington Group General Measure of Disability for older adults. Materials and Methods: This study used the 2012 California Health Interview Survey. The sample included 14,115 non-Hispanic White, Black, Hispanic and Asian adults aged 65 and older. Analysis was conducted using multi-group confirmatory factor analysis (CFA), parallel and Tau-equivalent tests. Results: The results indicated that the measure was valid for use with older adults (Satorra Bentler χ^2^ = 13.27, df = 3, *p* = 0.005, GFI = 0.996). Multi-group CFA indicated comparisons were valid between Whites with Blacks, and Hispanics with Asians. Cognitive disability was associated with independent living disability for Whites and Blacks, and with sensory disability for Hispanics and Asians. Conclusions: Findings indicated the measure is valid for cross-cultural comparison for certain racial/ethnic groups. Further research is needed to understand differences in associations of cognitive decline with other areas of disability for older adults.

## 1. Introduction

For more than a decade, the Washington Group General Measure has been used as a standardized set of questions to identify the prevalence of disability in population health surveys [1,2]. This measure was developed by the World Health Organization (WHO) to capture functional disability and included questions on sensory impairment and loss of functioning in domains of mobility, cognition, self-care, and independent living [3] (See Table 1). It was designed to be highly relevant for policy-makers and can be feasibly implemented by census bureaus across different countries [2]. Since its use, it has been found to be effective in identifying disability in populations, especially in domains most closely associated with social exclusion [4,5]. However, there is a gap in empirical research validating the Washington Group Measure for racially diverse older adults. Without validation, researchers may not be able to trust the accuracy of this measure in capturing loss of functioning for different racial and ethnic older adult populations.

Disability is a part of the aging process, and older adults experience loss of functioning in a continuum as they become older. A substantial body of research has identified differences in the aging process for non-Hispanic White older adults. Determining whether the Washington Group measure is cross-culturally equivalent is important for accurately identifying disability for minority older adult populations, which are rapidly growing. By 2050, it is estimated that 41% of older adults in the U.S. will be of Black, Hispanic, and Asian descent [6]. Additionally, findings from this measure are used by U.S. policy-makers to allocate resources for public health and community-based services [6]. Ensuring the accuracy of this measure is necessary for the appropriate allocation of targeted funding for racially diverse and underserved older adult populations. This will enable community-based agencies and providers to better identify and address the needs of minority older adults as they encounter loss of functioning in the aging process. 

### 1.1. Literature Review

#### 1.1.1. Minority Older Adults and Disability

The U.S. Census indicates that there are 44.7 million Americans who are 65 and older [6]. By 2050, this number will exceed 88.5 million, accounting for 20.1% of the total population. Over 31.2 million, or 12.6% of Americans over the age of 18, have a disability. Past scholarship highlighted that the likelihood of having a disability increases with age [7,8,9,10]. When comparing all age groups, non-Hispanic Whites (13.0%) and African Americans (13.9%) have higher rates of disability, compared to Hispanic (8.7%) and Asian Americans (6.9%) who tend to have younger average ages [11]. However, the prevalence of disability converges across racial and ethnic groups for older adults. At 65 to 74, the disability rate of Asians (19.5%) becomes closer to that of non-Hispanic Whites (25.0%), and the disability rate of Hispanics (31.0%) becomes closer to African Americans (34.2%). At 75 and older, the disability rate of Asians (49.9%) is statistically similar to non-Hispanic Whites (51.9%), and the disability rate of Hispanics (56.4%) is statistically similar to their African American counterparts (57.2%) [6].

#### 1.1.2. Conceptualization of Disability

The Americans with Disabilities Act of 1990 defines disability as a physical or mental impairment that substantially limits one or more major life activities [12]. Seminal scholars such as Verbrugge and Jette have conceptualized disability as the fundamental ability for a person to interact with the environment to meet their essential needs [13]. Disability is a multidimensional construct, and can be understood in functional, occupational, emotional, and social domains. Disability has been conceptualized and measured in the U.S. Census since 1830, and early surveys were based on the biomedical model with questions focused on sensory, mental, and physical deficits [14,15]. The framework of disability has evolved to include a focus on social and environmental factors, with regard to how persons can be supported to meaningfully function and fulfill societal roles [1] which is consistent with the conceptualization of disability from the United Nations Convention on the Rights of Persons with Disabilities (CRPD) [16]. More recent epidemiological studies examine disability in the context of overall health, functioning, and participation in social activities [4]. 

A sizeable body of literature has offered various definitions of disability. Verbrugge and Jette in 1994 have defined disability as difficulty performing activities of daily living due to health or physical problems [12]. This conceptual definition emphasizes the need for solutions to lessen the gap between personal capability and environmental demand by making accommodations and modifications to the environment. Further theoretical developments have applied the social model as a framework for understanding disability as a contextualized situation, and not as personal health limitations [17,18,19]. These developments have had an important impact on how disability has been examined in scholarship and empirical research, particularly in the epidemiology of disability and the prevention of further disablement of older adults. For the current study, disability is measured through the Washington Measure which is informed by impairment and loss of functioning as they relate to difficulties in meeting environmental demand.

#### 1.1.3. Measurement of Disability

Population health research primarily measures disability by examining domains related to sensory problems (seeing and hearing), cognitive impairments (memory and learning), ADLs (Activities of Daily Living) and IADLs (Instrumental Activities of Daily Living) [20]. These domains include questions regarding the ability to perform personal care, get around in the home, do chores, prepare meals, and run errands outside the home [11,20,21,22,23]. Examining these domains can provide useful information regarding successful aging in place and whether older adults can safely provide care for oneself and live independently. 

The current study examined the cross-cultural psychometric properties of the Washington Group General Measure of Disability (Table 1) from the California Health Interview Survey (CHIS). 

Although there exists a number of instruments that measure disability (i.e., Sheehan Disability Scale, Katz’s Activities of Daily Living Index), the Washington Group measure is one of the most commonly used in population health surveys due to its practicality and feasibility. Many different available sources for demographic analyses have examined disability using this measurement, such as the American Community Survey (ACS) from the U.S. Census, the Behavioral Risk Factor Surveillance System (BRFSS) from the Centers for Disease Control and Prevention, the Health and Retirement Study (HRS), the Medicare Current Beneficiary Survey (MCBS), the National Health Interview Survey (NHIS), and the National Health and Nutrition Examination Survey (NHANES) [20]. Because it is so widely-used, establishing its validity and cross-cultural equivalence for older adult populations is important for advancing research on disability for minority older adults. 

#### 1.1.4. Washington Group General Measure of Disability

The conceptual framework for the Washington Group Measure is based on the goal of equalization of opportunities from the World Health Organization (WHO) International Classification of Functioning, Disability, and Health (ICF). Past research has provided evidence that this measure is suitable for international comparisons [21]. It was constructed with input from representatives from over 100 countries, for the purposes of identifying persons with similar types and levels of basic activity limitations that can be applicable for all nationalities and cultures [24]. The measure captures disability for persons experiencing restrictions with performing everyday tasks and participating in employment [5], and works best at capturing the lowest levels of functioning [1]. However, the measure was not designed to capture the full spectrum of disability. This may be problematic for non-white older adults, who may underreport their loss of functioning because they have a different process of disablement compared to non-Hispanic White populations [25,26]. 

#### 1.1.5. Significance of Study

There are racial differences in the trajectory of aging for minority older adults, and these differences in the aging and disablement process can lead to non-equivalence in how disability is measured for White and non-White older adult populations. This study has two aims. First, the study aims to validate the Washington Group General Measure of Disability for a population-based sample of non-Hispanic White, Black, Hispanic and Asian adults 65 and older. The second aim is to determine the equivalence of the measure across these different older adult racial and ethnic groups. It was hypothesized that the psychometric properties of the Washington Group Measure will statistically vary across groups of non-Hispanic White, Black, Hispanic and Asian older adults. However, it is unclear if these differences would be found for all pairwise comparisons (i.e., non-Hispanic White vs. Black, non-Hispanic White vs. Hispanic, Non-Hispanic White vs. Asian, Black vs. Hispanic, Black vs. Asian, and Hispanic vs. Asian). 

## 2. Materials and Methods

### 2.1. Sample

This study used the 2012 California Health Interview Survey (CHIS) [27]. The CHIS data is the largest state survey on health and health characteristics, collected biennially since 2001 and annually beginning in 2013. Using a multistage sampling design, data was collected using random-digit-dialing (RDD) with landline and cellular samples to create a population-based representative sample of Californian adults. Interviews were conducted in five languages: English, Spanish, Chinese (Mandarin and Cantonese), Vietnamese, and Korean. The CHIS dataset is publicly available and therefore exempt from IRB approval.

The final study sample consisted of respondents age 65 and older (n = 14,115), with subgroups of non-Hispanic White (n = 10,662), Black (n = 601), Asian (n = 1201), and Hispanic (n = 900) older adults. These categories for race/ethnic group are based on those used in the U.S. Census.

### 2.2. Questions on the Washington Group Measure

The Washington Group General Measure of Disability consists of five self-report questions which captured (1) sensory disability, (2) cognitive disability, (3) self-care disability, (4) independent living disability, and (5) ambulatory disability (see Table 1). Respondents answered “yes” or “no” to the five questions in the measure. A response of “yes” was coded 1, and a response of “no” was coded 0. The yes/no construction of questions allowed for easier implementation and was designed to capture difficulty or limitations in domains of disability. No missing data was found for the measure items in the study sample.

### 2.3. Data Analysis

Descriptive analyses were conducted to examine differences in sociodemographic variables (gender, age category, poverty level, foreign-born status) and responses on disability questions for non-Hispanic White, Black, Hispanic and Asian older adults in the study. Descriptive and Kuder–Richardson reliability analyses were performed using Stata 15.0 (StataCorp, College Station, TX, USA) [28]; all further analyses were conducted in Lisrel 9.0 (Scientific Software International, Chapel Hill, NC, USA) [29]. Reliability and confirmatory factor analyses (CFA) were conducted for the full sample to determine the validity of the Washington Group Measure for all older adults included in this study. Separate confirmatory factor analysis models were examined to assess measurement validity for subgroups of non-Hispanic White, Black, Asian, and Hispanic older adults [30,31]. Parallel and Tau-equivalent models were statistically examined using structural equation modeling (SEM) across subgroups. This was performed to determine how well the measure captured a single, latent construct as a composite true score for each racial and ethnic group [32]. Measurement invariance analyses were conducted using multi-group confirmatory factor analysis techniques to determine cross-cultural equivalence when comparing the measurement with non-Hispanic White, Black, Asian, and Hispanic older adults. 

## 3. Results

### 3.1. Descriptive Statistics

Descriptive analyses are presented on Table 2 on sociodemographic variables for non-Hispanic White, Black, Hispanic and Asian older adults. Overall, six out of 10 older adult respondents were female. Non-Hispanic White and Black subgroups on average were older than Hispanic and Asian subgroups. More Hispanic and Asian older adults were below the Federal Poverty Level (Hispanics: 33.7%, Asians: 33.7%), compared to non-Hispanic White and Black respondents (non-Hispanic Whites: 5.8%, Blacks: 13.5%,). More Hispanics (59.1%) and Asians (85.6%) were foreign-born, compared to their non-Hispanic White (8.4%) and Black (3.7%) counterparts. Descriptive findings from this demographic analysis is representative of the specific race groups of older adults in the California population. 

From Table 3, the prevalence of older adults with at least one disability was highest for Hispanic and Black older adults, followed by non-Hispanic Whites and Asians (non-Hispanic White: 51.8%; Black: 55.2%; Hispanic: 59.3%; Asian: 47.1%). Regarding specific disability domains, ambulatory disability was most frequently reported across racial and ethnic groups (non-Hispanic White: 35.7%, Black: 42.1%, Hispanic: 40.1%, Asian: 25.4%). Black and Hispanic older adults have higher prevalences of self-care (Black: 12.8%, Hispanic: 11.0%) and independent living disability (Black: 15.8%; Hispanic: 14.9%). The prevalence of cognitive disability is higher for Blacks (22.5%), Hispanics (26.6%) and Asians (22.2%), compared to non-Hispanic Whites (18.5%). Hispanic and Asian older adults report more sensory disability (Hispanics: 21.0%, Asians: 20.5%), compared to their non-Hispanic White (17.2%) and Black (12.8%) counterparts.

### 3.2. Reliability Analysis Using Kuder–Richardson (KR20)

Kuder–Richardson reliability analysis indicated that the internal consistency of the Washington Group Measure was moderate for the full sample (KR20 = 0.58) (See Table 4). Internal consistency of the measure improved marginally in subgroup analyses for Black, Hispanic and Asian older adults (Black: KR20 = 0.62; Hispanic: KR20 = 0.63; Asian: KR20 = 0.63), and a small decrease was observed for non-Hispanic Whites (non-Hispanic White: KR20 = 0.56) (See Table 5).

### 3.3. Full Sample Confirmatory Factor Analysis

From Table 4, confirmatory factor analysis conducted on the measure for the full sample indicated a reasonably good fit (Satorra–Bentler scaled χ^2^ = 13.270, df = 3, *p* = 0.0049, CFI = 1.00, GFI = 0.996, AGFI = 0.98) (See Table 4). Self-care (λ = 0.89), independent living (λ = 0.85), and ambulatory disability (λ = 0.80) had the highest factor loadings, indicating that these domains most strongly represented disability in the measurement. Correlations in measurement errors were modeled for sensory disability with cognitive disability (θδ = 0.17) and sensory disability with independent living disability (θδ = 0.11). The unaccounted variability in sensory disability was associated with cognitive disability and independent living disability in the overall model.

### 3.4. Subgroup Confirmatory Factor Analysis

Separate CFA models with non-Hispanic White, Black, Hispanic and Asian older adult subgroups are presented in Table 5. Better goodness of fit statistics were observed for subgroup CFA models for Blacks, Hispanics and Asians compared to the model for non-Hispanic White older adults. 

#### 3.4.1. Non-Hispanic White Older Adults

Subgroup CFA analysis for the sample of non-Hispanic White older adults indicated moderate goodness of fit statistics (Satorra–Bentler scaled χ^2^ = 43.639, df = 4, *p* < 0.00001, CFI = 0.998, GFI = 0.984, AGFI = 0.938). Similar to results for the aggregate group (Table 4), self-care disability (λ = 0.89), independent living disability (λ = 0.83), and ambulatory disability (λ = 0.82) were most accounted for in the factor solution. Correlations in measurement errors were modeled for cognitive and independent living disability (θδ = 0.07) and sensory and ambulatory disability (θδ = 0.14), which improved the goodness of fit. Results indicated that the unaccounted variability in cognitive disability was associated with independent living, and sensory disability with ambulatory disability for non-Hispanic White older adults (see Table 5). 

#### 3.4.2. Black Older Adults

Subgroup CFA analysis for Black older adults indicated the model had a good fit (Satorra–Bentler scaled χ^2^ = 2.569, df = 4, *p* = 0.6323, CFI = 1.00, GFI = 0.982, AGFI = 0.932). Self-care disability (λ = 0.95), independent living disability (λ = 0.86), and ambulatory disability (λ = 0.84) were most accounted for in the factor solution. Correlations in measurement errors were modeled for cognitive disability and independent living disability (θδ = 0.15). Results indicated that the unaccounted variability in cognitive disability was associated with independent living disability for Black older adults (see Table 5). 

#### 3.4.3. Hispanic Older Adults

Subgroup CFA analysis for Hispanic older adults indicated a moderately good fit for the model (Satorra–Bentler scaled χ^2^ = 9.395, df = 4, *p* = 0.052, CFI = 0.998, GFI = 0.962, AGFI = 0.858). Self-care disability (λ = 0.91), independent living disability (λ = 0.90), and ambulatory disability (λ = 0.78) had the strongest factor loadings in the analysis. Factor loadings for sensory disability (λ = 0.49) were the strongest for Hispanic older adults compared to other groups. Correlations in measurement errors for cognitive disability and sensory disability (θδ = 0.11) were modeled to improve goodness of fit. Results indicated that the unaccounted variability in cognitive disability was associated with sensory disability for Hispanic older adults (see Table 5). 

#### 3.4.4. Asian Older Adults

Subgroup CFA analysis for Asian older adults indicated a very good fit in the model (Satorra–Bentler scaled χ^2^ = 2.35, df = 4, *p* = 0.67, CFI = 1.00, GFI = 0.992, AGFI = 0.970). Similar to other subgroups, self-care disability (λ = 0.85), independent living disability (λ = 0.94), and ambulatory disability (λ = 0.73) had the strongest factor loadings. Factor loadings for sensory disability (λ = 0.40) were stronger for Asian older adults compared to non-Hispanic Whites and Blacks. Asian older adults had the highest factor loadings for cognitive disability (λ = 0.58) compared to other subgroups. Correlations in measurement errors for cognitive and sensory disability were modeled (θδ = 0.32) to improve goodness of fit. Similar to their Hispanic counterparts, the unaccounted variability in cognitive disability was associated with sensory disability for Asian older adults (see Table 5).

### 3.5. Tests of Composite True Score of Disability

From Table 6, parallel models were examined to determine how well the disability questions produced a composite true score for different racial and ethnic groups of older adults. Measurement errors were modeled to be identical for all questions (see Table 6). Tests of parallel models examined whether the questions measure a single latent construct of disability, and that all questions individually measured disability with the same scalability, degree of precision and amount of measurement error [30,31]. In practice, disability is usually treated as a single latent construct, and each question is assumed to measure disability equally to form a composite true score. Goodness of fit results indicated an inadequate fit in the parallel models for all older adult subgroups, which suggest that there were differences in how questions in each domain measured disability in scalability, precision and accuracy. Factor loadings and measurement errors for self-care and independent living disability were similar across racial and ethnic subgroups. Factor loadings and measurement errors for cognitive disability were most similar for non-Hispanic Whites (λ = 0.51, δ = 0.54), Blacks (λ = 0.54, δ=0.52) and Hispanics (λ = 0.53, δ = 0.50). Factor loadings and measurement errors for sensory disability were most similar for Hispanic (λ = 0.57, δ = 0.50) and Asian (λ = 0.55, δ = 0.48) older adults, compared to other subgroups (see Table 6). 

From Table 7, Tau-equivalent models were conducted for all subgroups of older adults. The Tau-equivalent model shares many of the same assumptions as the parallel model and examines if questions measure a single latent construct of disability. Questions are tested to have the same scalability and degree of precision, but the amount of measurement error is allowed to vary within the model. Findings indicated that Tau-equivalent models for subgroups yielded an inadequate fit (Table 7). Similar factor loadings and measurement errors were observed for sensory disability with Hispanic (λ = 0.80, δ = 0.80) and Asians (λ = 0.78, δ = 0.85), cognitive disability for Blacks (λ = 0.81, δ = 0.88) and Hispanics (λ = 0.80, δ = 0.80), self-care disability for Blacks (λ = 0.81, δ = 0.21) and Hispanics (λ = 0.80, δ = 0.23), and independent living disability for Blacks (λ = 0.81, δ = 0.24), Hispanics (λ = 0.80, δ = 0.22), and Asian older adults (λ = 0.80, δ = 0.24).

### 3.6. Tests of Measurement Invariance

Tests of measurement invariance were conducted to determine equivalence in the Washington Group Measure when comparing non-Hispanic White, Black, Hispanic and Asian older adults. Similarities were found in measurement properties between non-Hispanic Whites and Blacks, and Hispanics and Asians in descriptive findings (Table 3), subgroup factor loadings (Table 5), error covariances (Table 5), measurement errors (Table 6 and Table 7), and goodness of fit indices (Table 5, Table 6 and Table 7). Based on previous findings, invariance tests were conducted to compare (1) non-Hispanic White and Black older adults and (2) Hispanic and Asian older adults. Cross-cultural measurement invariance tests on the Washington Group General Measure of Disability were based on the following two hypotheses: (1) The five-item disability scale is equivalent for non-Hispanic Whites compared to Blacks, and (2) the five-item disability scale is equivalent for Hispanics compared to Asians. For the first invariance hypothesis, results indicated that the Washington Group measure was equivalent for non-Hispanic White and Black older adults (Satorra-Bentler Adjusted χ^2^ = 4.80, df = 15, *p* = 0.78, RMSEA = 0.0797, NFI = 0.997, Critical N = 4101.925, GFI = 0.975). For the second invariance hypothesis, results indicated that the measure was equivalent for Hispanic and Asian older adults (Satorra-Bentler Adjusted χ^2^ = 15.09, df = 15, *p* = 0.06, RMSEA = 0.101, NFI = 0.996, Critical N = 2625.79, GFI = 0.969).

## 4. Conclusions

This study examined the measurement properties of the Washington Group General Measure of Disability with population-based data for non-Hispanic White, Black, Hispanic and Asian older adults. The findings suggested that the Washington Group Measure reasonably captured disability for the overall older adult population. However, the psychometric properties of the instrument were observed to differ among non-Hispanic White, Black, Hispanic and Asian older adults. Results indicated that the Washington Group Measure of Disability was statistically similar and measurement invariant for (1) Black and non-Hispanic White older adults, and (2) Hispanic and Asian older adults. Cross-cultural comparisons can be valid for comparing non-Hispanic White and Black older adults and comparing Hispanic and Asian older adults. Cognitive disability was associated with independent living disability for Blacks and non-Hispanic Whites. For Hispanics and Asians, cognitive disability was associated with sensory disability.

For non-Hispanic White, Black, Hispanic and Asian older adults, disability as defined by the Washington Group General Measure of Disability was most strongly represented by self-care, independent living, and ambulatory disability. Self-care, independent living, and ambulatory disability are important domains for consideration for older adults, especially in the context of aging in place. Although self-care and ambulatory disability had strong factor loadings for Asian older adults, there appears to be notable measurement error in these two questions, higher than for their non-Hispanic White, Black, and Hispanic counterparts (see Table 5). This may be explained by higher expectations of assistance in Asian cultures, where it is more acceptable and expected that family members would help out with activities such as dressing and bathing (self-care) and lifting and carrying (ambulatory) [33,34]. This may have contributed to the higher measurement errors in self-care and ambulatory disability for Asian older adults. In addition, Asian older adults are traditionally more likely to live in intergenerational households, where this type of assistance may be more readily available [34]. For Asian older adults, self-care and independent living disability may be mitigated by family supports [34], and can potentially be interpreted differently through a cultural lens.

Independent living disability accounted for a higher proportion of disability for Hispanics and Asians compared to their non-Hispanic White and Black counterparts. The ability to live independently requires interacting with the outside world to shop for food, see a doctor, or run errands [4,5]. The higher factor loadings for independent living disability for Hispanic and Asian older adults may be explained by the unique cultural and linguistic barriers which they encounter, as majority foreign-born populations (Hispanic: 59.1%, Asians: 85.6%). Foreign-born status for Asian and Hispanic older adults can exacerbate the impact of independent living disability if family support is not available [33,35].

Results from subgroup analysis indicated that for non-Hispanic White and Black older adults, cognitive disability is associated with independent living disability (see Table 5). Living independently involves navigating one’s surroundings, which may be impacted by memory loss and difficulty with concentration. For Hispanic and Asian older adults, findings from this study indicate that cognitive disability is associated with sensory disability (see Table 5). Hispanic and Asian older adults who lack formal education may depend more on their ability to visually identify landmarks and remember cues in their environment [34,35]. Similarly, it is possible that these older adults may understand English as it is spoken, though not as it is written. Over a quarter of the study sample of older Hispanic and Asian older adults do not have any college education (27.1%), which reflects overall sample characteristics in the U.S. [36]. Most have lived and worked in the U.S for over 15 years (64.9%), and almost half (48.1%) reported they do speak English well or not at all. Past research has highlighted the link of hearing loss with cognitive decline [37]. Hispanic and Asian older adults without formal education may rely heavily on having conversations with others in their own language to remain cognitively active. It is possible that Hispanic and Asian older adults with limited English ability may experience more rapid cognitive decline due to mental isolation. Providers who work with Hispanic and Asian older adults can ask more in-depth questions regarding how they are able to see, hear, and remember cues and landmarks, in order to understand their level of cognitive functioning and recommend interventions and resources to delay further decline. 

### 4.1. Strengths and Limitations

This study used large scale, population-based data to examine racial and ethnic differences in the measurement of disability, and is an important strength in the generalizability of this study. The Washington Group Measure is widely used in many different population health surveys, and findings from this study can have important implications for this reason. In addition, this study employed advanced statistical measurement methodology to a degree that has rarely been used to test equivalence in the measurement of disability across different racial and ethnic groups of older adults. 

It is important to note that there are limitations in this study. First, the data comes from a sample from California only, which may hinder the overall generalizability of results. However, the California Health Interview Survey is unique in that it has representative samples of diverse race groups (specifically Hispanic and Asian) which is difficult to find in any other geographic area. Because the purpose of the dataset is to assess the cross-cultural measurement invariance of the Washington Measure among diverse groups of older adults, we believe this data was best suited for this purpose. Second, Asian and Hispanic older adults were examined as pan-ethnic racial groups. There are important factors in culture, migration histories, and linguistic differences among Asian and Hispanic ethnic groups, which may need further examination with measurement invariance validation. Future research can disaggregate ethnic groups within the Asian and Hispanic populations. Third, there are other important measures of disability which were not examined in this study. Examples include the Katz Activities of Daily Living scale, which can be more sensitive to populations with high service needs [1]. However, this and other measures are less commonly available in population health surveys. More detailed information on functional levels and other social and environmental factors can also further contextualize findings from the Washington Group Measure [1]. For example, the question for “Do you have a condition that substantially limits one or more basic physical activities such as walking, climbing stairs, reaching, lifting, or carrying?” is used in the Washington Group Measure to capture ambulatory disability. This question was designed to capture the possible impact of conditions on functional abilities, but not the degree to which they will contribute to limitations in performing activities of daily living (ADLs) [5]. A follow-up question on how domains of disability captured by the Washington Group Measure impact ADLs can provide greater context through a cultural lens and align better with a social model framework of disability. 

### 4.2. Implications

Findings in this study highlighted the need for cross-cultural equivalence testing with measures of disability for different racial and ethnic populations of older adults. There are substantial differences in cultural practices, migration histories, languages and backgrounds, which impacted how disability is captured by the Washington Group measure, particularly for Asian and Hispanic older adults. Identifying culturally valid and relevant measurements of disability is important for population health research, without which can lead to misalignment and misallocation of resources. Findings from examining the cross-cultural reliability and validity of the Washington Group Measure indicated that minority older adults responded differently to questions regarding their disability in this study. Understanding differences in how older adults across racial and ethnic groups perceive their disability can lead to better identification of public resources for more focused, culturally-appropriate interventions to improve their quality of life. Future research can also take into account urban and rural residential settings which may impact access to care for minority older adults.

Past research has found that racial differences in disability diminish between non-Hispanic Whites and African Americans as they age, once controlling for socioeconomic factors [33,34]. However, it is important to note that disability rates are highest for Hispanic and Black older adults, and differences in how disability is measured were found across racial and ethnic groups. Findings suggest that Hispanic older adults may experience a different disablement process compared to non-Hispanic Whites [35,38]. The evidence of accelerated decline in the disablement process for Hispanic older adults suggest that issues of inequality similar to those experienced by African Americans may persist over the life course for this population [35,38]. Past research has highlighted the intersectional impact of structural racism on health for African Americans and Hispanics [39]. For Hispanic older adults, factors related to language, immigration status and other socioeconomic factors may further explain disparities in disability for this population [35,38,40]. 

Similar to Hispanic older adults, Asians have substantial subgroup variability [41] and likely a different disablement process [34]. More research is needed to include questions that measure disability which are invariant for all racial and ethnic groups of older adults, and can be used concurrently with the Washington Group Measure. It is important to re-evaluate the conceptualization and measurement of disability to be more inclusive of growing populations of diverse older adults, especially in the context of structural factors such as racism and other social determinants of health. 

For Black older adults, findings indicate that the perception of cognitive disability is tied to independent living disability. Outreach is needed to engage Black older adults in order to delay cognitive decline and extend aging-in-place for this population. Research has provided evidence that the population of Black older adults with a disability is steadily increasing and living longer [40]. Services required to address their needs will be extensive and long term [40]. Given the myriad challenges faced by Black older adults, appropriate measurements are needed to identify the prevalence of disability for this population. The Washington Group Measure is a reasonably good measure for Black older adults in that it captures domains of disability tied to social isolation. The use of this measure to capture the prevalence of these disabling conditions, which are higher for Black older adults, is necessary to ensure that they can be adequately addressed.

Public policy regarding funding for services should take into account that sensory disability is tied to cognitive disability for Asian and Hispanic groups. These older adults tend to have lower service utilization rates, and prevention initiatives related to cognitive decline in particular have under-engaged Asian older adults [42,43,44,45]. Providers working to engage Asian and Hispanic older adults to prevent cognitive decline may wish to consider the use of culturally appropriate interventions targeting those with vision and/or hearing loss. There is a need for more culturally and linguistically validated measurements of disability for researchers and providers to use with diverse older adult populations. This can lead to better identification of disabilities, to inform more focused, culturally-appropriate interventions and improve quality of life for increasingly diverse aging populations.

## Figures and Tables

**Table 1:** Disability domains from the Washington Group General Measure of Disability †.

1. Sensory Disability: Are you blind or deaf, or do you have a severe vision or hearing problem?
2. Cognitive Disability: Any difficulty learning, remembering, or concentrating?
3. Self-care Disability: Any difficulty dressing, bathing, or getting around inside the home?
4. Independent Living Disability: Any difficulty going outside the home alone to shop or visit a doctor’s office?
5. Ambulatory Disability: Do you have a condition that substantially limits one or more basic physical activities such as walking, climbing stairs, reaching, lifting, or carrying?

† From the 2011–2012 California Health Interview Survey (CHIS).

**Table 2:** Descriptive statistics of sample of non-Hispanic White, Black, Latino, and Asians 65 and over from 2011–2012 CHIS (n = 14,115).

	Non-Hispanic White (n = 10,662)	Black (n = 601)	Hispanic (n = 900)	Asian (n = 1201)
**Sociodemographics †** Gender				
Male	38.5%	35.1%	38.9%	42.9%
Female	61.5%	64.9%	61.1%	57.1%
Age Category				
65 to 74	49.8%	54.7%	61.9%	55.5%
75 to 84	35.5%	33.9%	31.4%	36.0%
85 and older	14.7%	11.3%	6.7%	8.5%
Poverty				
Below Federal Poverty Level	5.8%	13.5%	33.7%	33.7%
Foreign-born Status				
Not Foreign-born	91.6%	96.3%	40.9%	14.4%
Foreign-born	8.4%	3.7%	59.1%	85.6%

† Reported percentages for all sociodemographic variables are significant at *p* < 0.01.

**Table 3:** Descriptive statistics of Washington Group General Measure of Disability for non-Hispanic Whites, Blacks, Hispanics, and Asians 65 and older from 2011–2012 CHIS (n = 14,115).

Disability Domains †	Non-Hispanic White (n = 10,662)	Black (n = 601)	Hispanic (n = 900)	Asian (n = 1201)
Sensory Disability	17.2%	12.8%	21.0%	20.5%
Cognitive Disability	18.5%	22.5%	26.6%	22.2%
Self-care Disability	7.5%	12.8%	11.0%	9.3%
Independent Living Disability	11.0%	15.8%	14.9%	11.4%
Ambulatory Disability	35.7%	42.1%	40.1%	25.4%
Have 1 or more Disability	51.8%	55.24%	59.3%	47.1%

† Reported percentages for disability are significant at *p* < 0.01 based on ANOVA.

**Table 4:** Confirmatory factor analysis model of Washington Group Measure for all non-Hispanic Whites, Blacks, Hispanics and Asians 65 and older from 2011–2012 CHIS (n = 14,115).

Disability Domains	λ (δ)
Sensory Disability	0.31 (0.90)
Cognitive Disability	0.43 (0.81)
Self-care Disability	0.89 (0.12)
Independent Living Disability	0.85 (0.28)
Ambulatory Disability	0.80 (0.36)
Error Covariance (θδ)	
Sensory and Cognitive Disability	0.17
Sensory and Ambulatory Disability	0.11
Cronbach’s Alpha α (Kuder–Richardson)	0.58
**Goodness of Fit Statistics †**	
Satorra–Bentler scaled χ^2^ (df)	13.270 (3)
*p*-Value	0.0049
RMSEA	0.016
NFI	1.00
NNFI	0.999
CFI	1.00
Critical N	12069.425
Standardized RMR	0.0132
GFI	0.996
AGFI	0.980

† RMSEA = root mean squared error of approximation, NFI = normed fit index, NNFI = non-normed fit index, CFI = comparative fit index, GFI = goodness of fit index, AGFI = adjusted goodness of fit index.

**Table 5:** Subgroup confirmatory factor analysis models: factor loadings and measurement errors of Washington Group Measure of Disability for non-Hispanic Whites, Blacks, Hispanics and Asians 65 and older from 2011–2012 CHIS (n = 14,115).

Disability Domains	Non-Hispanic Whites (n = 10,662) λ (δ)	Blacks (n = 601) λ (δ)	Hispanics (n = 900) λ (δ)	Asians (n = 1201) λ (δ)
Sensory Disability	0.29 (0.92)	0.25 (0.94)	0.49 (0.76)	0.40 (0.84)
Cognitive Disability	0.40 (0.84)	0.38 (0.86)	0.43 (0.82)	0.58 (0.67)
Self-care Disability	0.89 (0.20)	0.95 (0.11)	0.91 (0.18)	0.85 (0.27)
Independent Living Disability	0.83 (0.32)	0.86 (0.27)	0.90 (0.20)	0.94 (0.13)
Ambulatory Disability	0.82 (0.33)	0.84 (0.29)	0.78 (0.40)	0.73 (0.47)
Error Covariance (δθ)				
Cognitive and Sensory Disability			0.11	0.32
Cognitive and Independent Living Disability	0.07	0.15		
Ambulatory and Sensory Disability	0.14			
Cronbach’s Alpha α (Kuder–Richardson)	0.56	0.62	0.63	0.65
**Goodness of Fit Statistics †**				
Satorra–Bentler scaled χ^2^ (df)	43.639 (4)	2.569 (4)	9.395 (4)	2.35 (4)
*p*-value	<0.00001	0.6323	0.052	0.67
RMSEA	0.102	0.0981	0.152	0.06
NFI	0.998	0.998	0.996	0.999
NNFI	0.996	1.002	0.994	1.001
CFI	0.998	1.000	0.998	1.00
Critical N	3244.524	3101.676	1271.442	6768.147
Standardized RMR	0.0446	0.0388	0.0375	0.0153
GFI	0.984	0.982	0.962	0.992
AGFI	0.938	0.932	0.858	0.970

† RMSEA = root mean squared error of approximation, NFI = normed fit index, NNFI = non-normed fit index, CFI = comparative fit index, GFI = goodness of fit index, AGFI = adjusted goodness of fit index.

**Table 6:** Subgroup parallel models factor loadings and measurement errors of Washington Group General Measure of Disability for non-Hispanic Whites, Blacks, Hispanics and Asians 65 and older from 2011–2012 CHIS (n = 14,115).

Disability Domains	Non-Hispanic Whites (n = 10,662) λ (δ)	Blacks (n = 601) λ (δ)	Hispanics (n = 900) λ (δ)	Asians (n = 1201) λ (δ)
Sensory Disability	0.45 (0.54)	0.36 (0.52)	0.57 (0.50)	0.55 (0.48)
Cognitive Disability	0.51 (0.54)	0.54 (0.52)	0.53 (0.50)	0.69 (0.48)
Self-care Disability	0.78 (0.54)	0.82 (0.52)	0.82 (0.50)	0.79 (0.48)
Independent Living Disability	0.80 (0.54)	0.82 (0.52)	0.83 (0.50)	0.82 (0.48)
Ambulatory Disability	0.76 (0.54)	0.80 (0.52)	0.74 (0.50)	0.71 (0.48)
**Goodness of Fit Statistics †**				
Satorra–Bentler Scaled χ^2^ (df)	895.958 (9)	2.569 (9)	109.484 (9)	110.538 (9)
*p*-value	<0.0001	<0.0001	<0.0001	<0.0001
RMSEA	0.260	0.0981	0.281	0.264
NFI	0.962	0.998	0.954	0.967
NNFI	0.959	1.002	0.953	0.967
CFI	0.963	1.000	0.958	0.970
Critical N	258.810	3101.676	178.909	236.212
Standardized RMR	0.141	0.0388	0.129	0.114
GFI	0.823	0.982	0.807	0.794
AGFI	0.705	0.932	0.679	0.656

† RMSEA = root mean squared error of approximation, NFI = normed fit index, NNFI = non-normed fit index, CFI = comparative fit index, GFI = goodness of fit index, AGFI = adjusted goodness of fit index.

**Table 7:** Tau-equivalent model factor loadings and measurement errors of Washington Group General Measure of Disability for non-Hispanic Whites, Blacks, Hispanics and Asians 65 and older.

Disability Domains	Non-Hispanic Whites (n = 10,662) λ (δ)	Blacks (n = 601) λ (δ)	Hispanics (n = 900) λ (δ)	Asians (n = 1201) λ (δ)
Sensory Disability	0.75 (0.95)	0.81 (--)^a^	0.80 (0.80)	0.78 (0.85)
Cognitive Disability	0.75 (0.86)	0.81 (0.88)	0.80 (0.88)	0.78 (0.60)
Self-care Disability	0.75 (0.31)	0.81 (0.21)	0.80 (0.23)	0.78 (0.30)
Independent Living Disability	0.75 (0.30)	0.81 (0.24)	0.80 (0.22)	0.78 (0.24)
Ambulatory Disability	0.75 (0.36)	0.81 (0.28)	0.80 (0.40)	0.78 (0.47)
**Goodness of Fit Statistics †**				
Satorra–Bentler Scaled χ^2^ (df)	665.302 (9)	58.359 (9)	73.648 (9)	95.311 (9)
*p*-Value	<0.0001	<0.0001	<0.0001	<0.0001
RMSEA	0.220	0.257	0.221	0.228
NFI	0.972	0.961	0.969	0.972
NNFI	0.969	0.963	0.970	0.972
CFI	0.972	0.967	0.973	0.974
Critical N	348.191	223.759	265.477	273.789
Standardized RMR	0.212	0.256	0.209	0.174
GFI	0.831	0.787	0.838	0.824
AGFI	0.718	0.645	0.730	0.707

† RMSEA = root mean squared error of approximation, NFI = normed fit index, NNFI = non-normed fit index, CFI = comparative fit index, GFI = Goodness of fit index, AGFI = adjusted goodness of fit index. ^a^. The measurement error for sensory disability with Black older adults was 1.16, and thus unstable and not included in the tables.

## Data Availability

Data can be downloaded through the California Health Interview Survey portal.
